# Current Level and Correlates of Traditional Cooking Energy Sources Utilization in Urban Settings in the Context of Climate Change and Health, Northwest Ethiopia: A Case of Debre Markos Town

**DOI:** 10.1155/2014/572473

**Published:** 2014-05-07

**Authors:** Kumlachew Geremew, Molla Gedefaw, Zewdu Dagnew, Dube Jara

**Affiliations:** ^1^Migibare Senay Children and Family Support Organization, P.O. Box 269, Debre Markos, Ethiopia; ^2^Dean of GAMBY College of Medical Sciences, P.O. Box 79, Bahir Dar, Ethiopia; ^3^Department of Public Health, College of Medicine and Health Science, Debre Markos University, P.O. Box 269, Debre Markos, Ethiopia

## Abstract

*Background.* Traditional biomass has been the major source of cooking energy for major segment of Ethiopian population for thousands of years. Cognizant of this energy poverty, the Government of Ethiopia has been spending huge sum of money to increase hydroelectric power generating stations. *Objective.* To assess current levels and correlates of traditional cooking energy sources utilization. *Methods.* A community based cross-sectional study was conducted employing both quantitative and qualitative approaches on systematically selected 423 households for quantitative and purposively selected 20 people for qualitative parts. SPSS version 16 for windows was used to analyze the quantitative data. Logistic regression was fitted to assess possible associations and its strength was measured using odds ratio at 95% CI. Qualitative data were analyzed thematically. *Result.* The study indicated that 95% of households still use traditional biomass for cooking. Those who were less knowledgeable about negative health and environmental effects of traditional cooking energy sources were seven and six times more likely to utilize them compared with those who were knowledgeable (AOR (95% CI) = 7.56 (1.635, 34.926), AOR (95% CI) = 6.68 (1.80, 24.385), resp.). The most outstanding finding of this study was that people use traditional energy for cooking mainly due to lack of the knowledge and their beliefs about food prepared using traditional energy. That means *“…people still believe that food cooked with charcoal is believed to taste delicious than cooked with other means.”* *Conclusion.* The majority of households use traditional biomass for cooking due to lack of knowledge and belief. Therefore, mechanisms should be designed to promote electric energy and to teach the public about health effects of traditional cooking energy source.

## 1. Introduction

Energy for cooking usually constitutes 70% to 90% of total energy use in less industrialized countries [1]. It has been estimated that about 2.5 billion people in these countries rely on biomass fuels (such as firewood, charcoal, and animal dung) to meet their cooking energy needs. According to the International Energy Agency, 2010, without a substantial change in policy, the total number of people relying on biomass fuels will increase from today's 2.4 billion to 2.7 billion by 2030 [2].

According to a World Bank report, indoor air pollution in developing countries is designated as one of the four most critical global environmental problems [3–5]. Burning biomass fuel indoor is a major source of large amounts of smoke and other pollutants in the confined space of the home, thereby providing a perfect avenue for human exposure. In rural areas of Africa, a substantial portion of infants, children, and women is exposed to debilitating levels of indoor pollution caused by biomass fuel use, which has an inefficient combustion process and a very high particulate matter emission [6].

Concerning cooking energy consumption pattern, a report by the Ethiopian Rural Energy Development and Promotion Center (1998) revealed that 77% of total final energy consumption consisted of firewood and charcoal while another 15.5% consisted of agricultural residues; only roughly 6% was met by modern energy sources such as petroleum and electricity and only 1% of the population utilized electricity for cooking. In view of the increasing population, escalating prices, and shrinking reserves of oil and coal, adverse health effects of cooking energy sources is the current burning issue of the globe. In this regard, World Health Organization estimates that 1.5 million premature deaths per year are directly attributable to indoor air pollution (IAP) from the use of solid fuels [6].

Urban and rural communities in Ethiopia depend mainly on the use of traditional fuels like wood, dung, leaves, twigs, corncobs, charcoal, and other biomass fuels. The use of these fuels will lead to substantial air pollution problem caused by carbon monoxide, hydrocarbons, and particulate matters. These gases pollute the breathing air of those persons who are near the fireplace [4, 7].

On the other hand, using various types of wood and dung cake for cooking purpose is not only harmful for health but also one of the major causes of environmental pollution and energy crisis [8]. Today, the demand for cooking fuels, especially in urban areas, is advancing deforestation in hundreds of kilometers from the nearest city. Many of the poorest countries are severely deforested at rates approaching 95% and even 98%; in Africa it is responsible for over 90% of the woody biomass harvested [1].

A study done in Ethiopia in 2012 showed that about 77% of annual biomass consumption in Ethiopia is met from fuel wood followed by animal dung (13%) and crop residue (9%), respectively. Concerning regional distribution of biomass consumption, annually about 88% of total biomass fuel is consumed mainly in three regions: Amhara (34%), Oromia (32%), and SNNP region (22%). Similar study in southern Ethiopia reported that biomass fuels are the major sources of energy consumption. For more than 90% of the Ethiopian population, the only energy used for cooking is obtained from biomass, in which 99% is derived from fuel wood, charcoal, crop residue, and leaves, with fuel wood occupying the leading position. According to another study conducted in southern part of Ethiopia, the common domestic energy sources used for cooking and heating in all houses surveyed in the rural communities were biomass fuels such as wood, cow dung, and leaves corncobs. It was also found in the great majority of households that 72.5% do not have a separate kitchen and 92% did not have windows for ventilation [3, 9].

In addition to the above mentioned facts, cost of cooking energy is worsening communities' way of life. Despite the multidimensional impacts of household cooking energy sources on health, environment, and cost, adequate data pertinent to the subject matter is not available. Presently, there is little data documented in Ethiopia on the current level and correlates of utilization of traditional cooking energy sources among households. Therefore, producing more data related to household cooking energy sources is believed to fill the existing knowledge gap. The present study is conducted to determine current level and correlates of utilization of traditional cooking energy sources among households of Debre Markos town, northwest Ethiopia.

**Figure 1:**
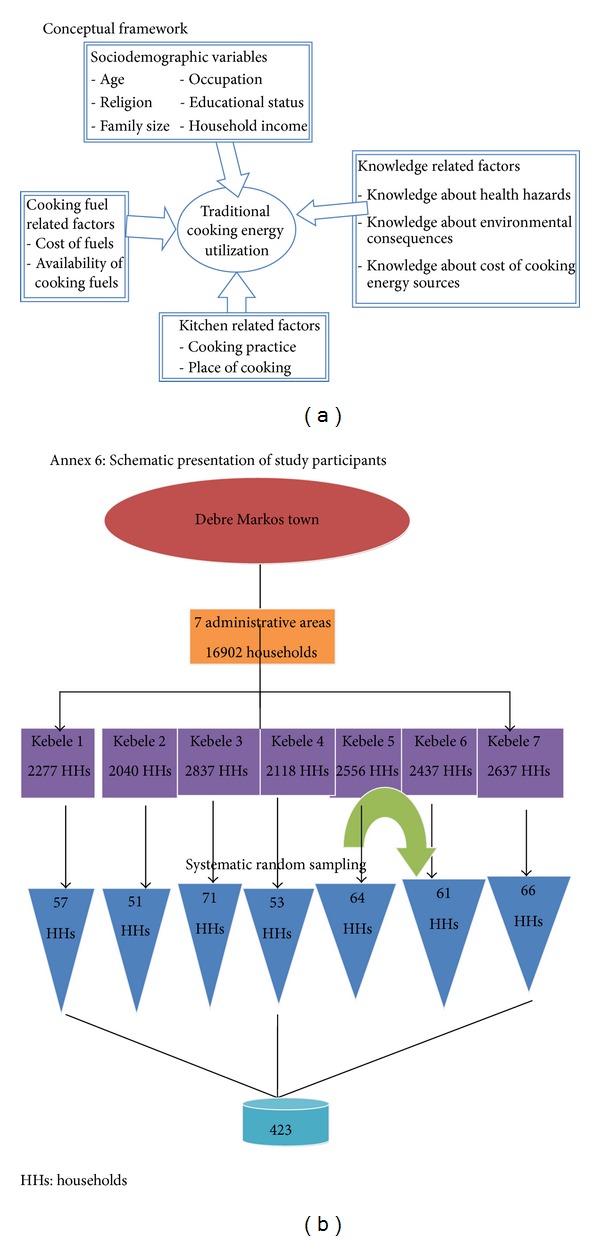
(a) Conceptual framework of factors affecting traditional cooking energy utilization, Debre Markos, 2013. (b) Schematic presentation of sampling procedure, Debre Markos, 2013.

Therefore, this study finding will have both theoretical and practical significance to fill the prevailing knowledge gap of practitioners, policy makers, program managers, funders, the local community, and researchers as well as to scale up modern energy source utilization (Figure 1).

## 2. Methods and Materials

### 2.1. Study Area and Setting

The study was conducted from January to March 2013 in Debre Markos town found in East Gojjam Zone of Amhara Regional State, Ethiopia. Debre Markos town is situated at an altitude of 2509 meters above sea level and located at 299 kms from Addis Ababa in the northwest of the country. The climatic condition of the town is temperate and cold. Its annual average temperature is 18.5°C. Regarding population and settlement, according to Debre Markos University official website at present, the total number of people in Debre Markos town is estimated to be 107,684 of which males are 49,893 and females are 57,791. The town is divided into 7 administrative kebeles. All kebeles of Debre Markos town were included in the study.

### 2.2. Study Design and Population

A cross-sectional study was conducted using mixed data collection method (quantitative study supplemented by qualitative method). Source population were all households in all kebeles of Debre Markos town for quantitative method and all experts who were working in cooking energy source related sectors and all government employed women who had better experience, knowledge, and belief about traditional cooking energy utilization for qualitative methods. Study population were all women resided in the randomly selected administrative areas of town for quantitative method and purposively selected experts who were working in EEPCO, in East Gojjam Zone mining and energy office and in GIZ during the study period and employed women who were living in the study area for qualitative methods. All registered households resided in the selected kebeles were included in the study. Apartments and public institutions were excluded from the study.

### 2.3. Sample Size Determination and Sampling Techniques

The required sample size was calculated using single population proportion formula by considering 50% proportion of traditional cooking energy utilization since there was no previous study in the same setting to the understanding of investigators, 5% margin of error and 95% CI were used for quantitative part. By adding 10% nonresponse rate, the final sample size was 423.* For qualitative part*, two experts from Ethiopian Electric Corporation Debre Markos District, 2 experts from Water, Mineral and Energy offices of East Gojjam Zone, and 16 government employed women from the community, a total of 20 individuals, were purposively selected for key informant interview and for an in-depth interview, respectively.

**Figure 2:**
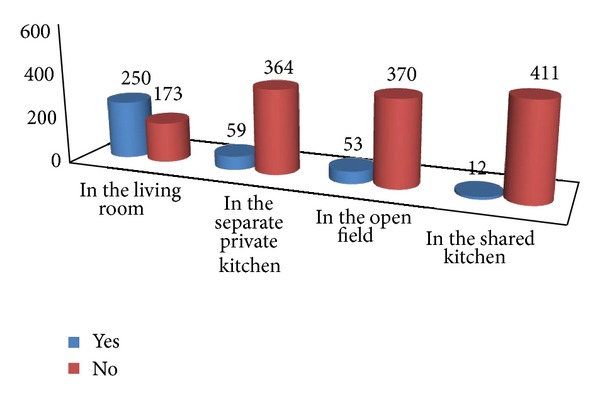
Responses of study participants on their place of cooking in Debre Markos town, Ethiopia, May 2013.

Systematic random sampling technique was used to select study participants for this study. The total number of households was identified through reviewing records in each kebele administrative office. Then, the total number of households was divided into the required sample size in each kebele proportional to the size of household. The first household was selected by lottery method and since the sampling interval was too great, creating large distances for surveyors to travel between houses, then the subarea and the sampling interval were divided by the same factor. Based on this, a sampling interval of every 8th household was visited to get the required number of study subjects in each kebele. Lottery method was used to select a woman if there were two or more women in a house (Figure 2).

### 2.4. Variables and Measurement

The dependent variable of the study was traditional cooking energy utilization and the independent variables were sociodemographic variables, kitchen related factors, knowledge related factors, and cooking fuel related factors. Traditional cooking energy sources include energy sources obtained from dung, agricultural residues, leaves, straw, wood charcoal, and fuel wood. Traditional cooking energy utilization: a respondent was considered as utilizing traditional cooking energy sources when she utilized firewood, charcoal, and animal dung for cooking purposes.* High utilization* is considered when the utilization rate is >85%,* moderate utilization* is considered when the utilization rate is 70–85%, and* low utilization* is considered when the utilization rate is <70%.* Modern cooking energy sources* are energy sources obtained from liquefied Petroleum gas, biogas and electricity, whereas traditional cooking energy sources: energy sources obtained from wood, charcoal, animal dung, straw, and leave.

### 2.5. Data Collection Instrument and Procedures

Quantitative data were collected using a structured interview questionnaire by 8 trained data collectors assisted by two supervisors. Collection was started after they had been given one-day training on data collection tools and procedures by the principal investigator. Qualitative data were collected through key informant interview and in-depth interview using unstructured and open-ended questionnaires in order to provide more insight into the reasons why many households were utilizing traditional cooking energy sources and why others were not.

### 2.6. Data Quality Assurance

Training was given to data collectors and supervisors by the principal investigator. The questionnaire was translated into Amharic language and translated back into English language to ensure the comparability of results. The questionnaire was pretested on 5% of the sample size out of the study area on similar population and corrections on the instruments were made accordingly. There was a meeting with data collectors to discuss the daily data collection procedures. For the qualitative data, notes were taken during each in-depth interview and in addition, a tape recorder was used to safeguard against the loss of information. The overall activity was controlled by the principal investigator.

### 2.7. Data Processing and Analysis

The quantitative data were checked for completeness and consistency. First descriptive analysis was carried out to explore the sociodemographic characteristics of the respondents. Bivariate analysis was used primarily to check which variables had association with the dependent variable individually. Variables were entered into multivariable logistic regressions for controlling the possible effects of confounders. Finally, variables which had significant association were identified on the basis of OR, with 95% CI and with *P* value ≤ 0.05 to fit into the final regression model. The qualitative data were summarized manually and thematically. The findings were supplemented during write-up.

Ethical clearance was obtained from Debre Markos University, Medicine and Health Sciences Ethical Review Committee. Verbal informed consent was secured from study subjects. Each respondent was informed about the objective of the study and assured of confidentiality of the information they provided. In order to protect confidentiality, names or actual house numbers were not included in the written questionnaires.

## 3. Result

### 3.1. Sociodemographic Characteristics of the Respondents

Four hundred twenty-three (423) women participated with overall response rate of 100%. Of those, 256 (60.5%) of the respondents were belong to age group of 25 years & above with median age of 38 years (IQR ± 22). One hundred fifteen (27%) of the respondents were unable to read and write, 42 (10%) attended primary school and 191 (45%) had completed high school and higher education. While the remaining 78 (8%) were able to read and write only. Almost all, 413 (97.6%), of the respondents were orthodox Christian followers. The majority, 273 (64.5%), of the respondents were ever married. The monthly median income of the respondents was birr 1000 (IQR ± 1300). One hundred sixty-six (39%) of the respondents had an income of 1001 birr and greater, while 257 (61%) of them reported that their monthly income was below 1001 birr. Alternatively, the income means for 96 (22.7%) of households were selling and buying goods and services, for 105 (25%) of them were government employment, and for the rest, 61 (14.41%) of the households, were self-employment. About 233 (55%) of respondents had family size of 2–4, whereas the remaining 190 (45%) had a family size of above 4 (Table 1).

### 3.2. Traditional Cooking Energy Utilization

According to this study, almost all, 403 (95%), of the respondents reported that they were utilizing traditional cooking energy sources. As reported by an expert from the mining and energy of East Gojjam administrative zone, people of this town heavily relied on traditional cooking energy sources thinking that these were the cheapest sources than the modern ones. But the reality is not that.

*“…the consequences of using traditional biomass fuels like charcoal for example are multifaceted; financial, environmental and health consequences can be mentioned. To impede these, environmentally friendly, health benefiting and cost effective stoves should be introduced by the government and non government organizations as being done by the likes of GIZ.”*  A 40-year-old woman when asked why many households were not utilizing cheap clean energy sources instead of the traditional ones, she replied,  *“these days we hear about such energy sources are cheaper than the traditional ones. But, they require huge investment cost that cannot be afforded by households like me.”*

### 3.3. Housing and Kitchen Characteristics

The kitchen characteristics in the majority of the households were remarkably similar. As shown in Figure 2, about 59.2% of the households of the samples had separate kitchen. More than half of the households (52.7%) had separate indoor kitchens outside the house with one window and with ventilation conditions; otherwise, in 25 (6%) of the households, the kitchens were found attached to the living houses. About 250 (59.1%) of the respondents were cooking in their living room (Figure 2).

**When a 36-year-old woman was asked where she usually cooked, she replied,*“place of cooking depends on the weather condition. In the winter season, we usually cook in the open field; whereas, in the summer seasons we will be restricted to cook inside just in the kitchens if we have or in the parts of our residences. While we are cooking with charcoal we usually use our residence as proper place of cooking.” *

### 3.4. Patterns of Household Cooking Energy Utilization

**Figure 3:**
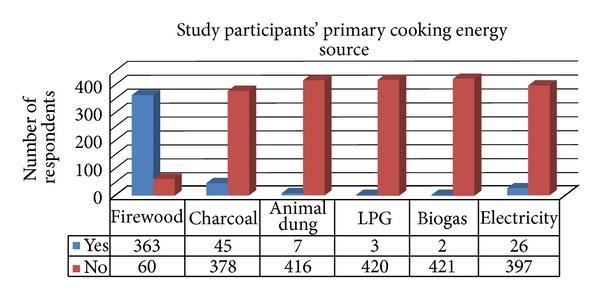
Primary cooking energy sources of study participants among households in Debre Markos town, northwest Ethiopia, May 2013.

As shown in Figure 3, most, 363 (85.8%), of the respondents reported that firewood was used as their primary cooking source and 45 (10.6%) of them used charcoal as primary cooking energy source. Electricity as a primary cooking energy source was mentioned only by 26 (6.1%) of the respondents. Only 3 (0.7%) of the respondents preferred using LPG for cooking their food while the remaining 2 (0.5%) and 7 (1.7%) of them mentioned biogas, animal dung, and leaves as primary cooking energy sources, respectively.

A 46-year-old woman working in a government organization reported* “my household mostly depends on fuel-wood for our cooking purpose. The reason for our choice of fuel-wood is closely linked to its availability as compared with other sources.”* She added* “fuel-wood cooks faster than kerosene while biogas for cooking is unpopular in our community.”* An expert from the GIZ reported* “using clean energy sources for cooking purposes saves time, easy to use, no negative health effects, despite it has high initial investment costs.”*

### 3.5. Effects of Cost of Cooking Energy Sources on Traditional Cooking Energy Utilization

The mean cost of fuels for cooking wote once a day and mean cost of fuels for baking injera once a week were found to be birr 10.75 (SD ± 2.704) and birr 21.56 (SD ± 2.77), respectively. Furthermore, as shown in Table 2 below, most of the respondents, 416 (98%), reported that they were using firewood. Accordingly, 318 (75.2%) of those who were utilizing firewood were paying birr 8 to birr 100, whereas 105 (24.8%) of the respondents were paying birr 101 to birr 500. Only 1 respondent (0.2%) was paying more than birr 501. On the contrary, respondents who were utilizing electricity were only 36 (8.5%). Based on this, 29 (7%) of the respondents were paying birr 10 to birr 75 on monthly basis and the remaining 7 (1.7%) of the respondents paid birr 76 to birr 100 (Table 2). According to an expert from EEPCO, people in Debre Markos town have a cultural attachment to cooking with wood, animal dung, leaves, and charcoal.

*“…the government is working hard to promote electricity use for household cooking. In the last few years for example, there has been a large increase in grid connections, however, people still choose not to use electricity for cooking purposes.”*

On the other hand, most of the interviewed employed women reported that using electricity for cooking requires consistent connection. A 33-year-old woman reported* “most of the time while we bake Injera for example electric breakdown was happening that disrupted the process.”*

### 3.6. Respondents' Knowledge on Health, Environmental, and Cost Effects of Traditional Cooking Energy Utilization

As shown in Table 3, out of the total women who responded to have knowledge of health effects of traditional cooking energy sources, only 178 (42.1%) were able to name most of the accepted traditional cooking energy utilization related health problems such as cough, irritation of eyes, and breathing related problems. Regarding the respondents' multiple responses about their knowledge of the type of cooking energy sources that cause health problems, 89% of the respondents mentioned smoke from charcoal, 91% of the respondents said smoke from firewood, and 89% of the respondents reported that smoke from animal dung and leaves disrupt one's health (Table 3).

Respondents were asked whether they knew that smoke from firewood, animal dung, leaves, and charcoal affects the environment. As shown in Table 3 below, half (50.6%) of respondents said that they knew about it, while mentioning most accepted environmental consequences resulting from excessive fuel wood consumption, about 329 (78%) of them mentioned deforestation, erosion, pollution, lack of oxygen, and wild life destruction.

Furthermore, respondents were asked whether they could relate traditional cooking energy utilization to the most common health problems. Based on this, 353 (83.5%), 390 (92.2%), and 172 (40.7%) respondents related traditional cooking energy utilization to cough, to irritation of the eyes, and to sinus, respectively.

### 3.7. Availability of Cooking Energy Sources

**Figure 4:**
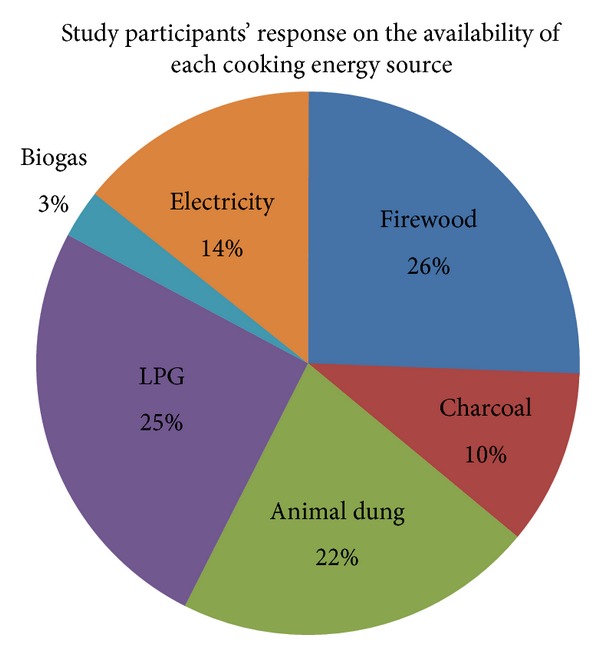
Responses of participants on the availability of cooking energy sources in Debre Markos town, Ethiopia, April 2013.

With regard to the availability of cooking energy sources, almost all, 303 (74%), of respondents agreed that wood, charcoal, animal dung, and leaves were easily available, whereas only 110 (26%) of study participants reported that biogas was easily available for cooking. An expert from the mining and energy bureau was asked about the energy alternatives available for cooking. According to the expert, both modern and traditional cooking energy alternatives were available for the community (Figure 4).

*“…though clean energy sources are becoming available for the households; firewood and charcoal are the primary means for cooking even in the urban areas of Debre Markos town.”*  The expert gave the following reasons for this trend.**

*“…people of Debre Markos town has been using charcoal, firewood and animal dung for hundreds and hundreds of years. It was not what they know and what they were comfortable with to switch to modern sources.”*

*“…still food cooked with charcoal for example is believed to taste delicious than when cooked with other means.”*

### 3.8. Correlates of Traditional Cooking Energy Utilization

To find out correlates of traditional cooking energy utilization, bivariate and multivariable analysis were conducted considering sociodemographic variables, knowledge related variables, fuel related variables, and sociocultural variables as independent variables and traditional cooking energy utilization as dependent variable. In bivariate analysis, income group, knowledge about effects of firewood smoke on health, knowledge about effects of wood, charcoal, dung, and leaves' smoke on the environment, effect of cost on fuel choice, and availability of cooking energy sources were found to be associated with traditional cooking energy utilization.

Multivariable analysis based on backward stepwise logistic regression also showed that knowledge about the effects of firewood smoke on health, knowledge about the effects of firewood smoke on the environment, income, availability of cooking energy sources, and effect of cost on fuel choice (expense perception) had an association with traditional cooking energy utilization.

Knowledge about effects of firewood smoke on health had shown statistically significant association with traditional cooking energy utilization. Households who had less knowledge about the effects of firewood smoke on health were 7.56 times more likely to utilize traditional cooking energy sources as compared with those who had sufficient knowledge about them with (AOR = 7.56 (95% CI, 1.635, 34.926)).

Statistical significant association was also found between respondents' knowledge about effects of firewood, charcoal, dung, and leaves' smoke on the environment and traditional cooking energy utilization. Women who had less knowledge about the effects of firewood, charcoal dung, and leaves' smoke on the environment were 6.68 times more likely to utilize traditional cooking energy sources than those who had sufficient knowledge about them with (AOR = 6.68 (95% CI, 1.80, 24.385)).

When asked how people could be educated to shift to modern fuels, the expert from the mining and energy office said

*“…it will be possible to motivate people of this town to shift to modern fuels; however, it will require much effort from both government and other non government organizations in the promotion of such fuels.”*  The following points were proposed by this expert.**

*“…people need to be educated about the dangers associated with traditional biomass use; they need to understand the benefits of modern stoves as well.”*

*“…people need to understand that when using traditional biomass fuels, trees have been cut; this tree that serves many functions as ecological imbalance, fruit bearing, shade creating, and many more.”*

According to the expert from the mining and energy bureau of East Gojjam administrative zone, it is important to mobilize different government and nongovernment factors for the promotion of modern fuels.

**A 25-year-old woman when asked about the effects of traditional cooking energy sources on health, environment, and the economy, she reported*“I know that smoke from firewood, charcoal, and animal dung affects our health irrespective of what specific problems can results. I do not think that traditional cooking energy sources are cheaper than the modern ones.”*She added that*“media is reporting that smoke from any source is affecting our planet, but I do not know how it is happening.”*

Availability of cooking energy sources was an important predator of traditional cooking energy utilization. Respondents who said wood, charcoal, and animal dung were easily available were 3.03 times more likely to utilize traditional cooking energy sources as compared with those who said electricity, LPG, and biogas were easily available (AOR = 2.76 (95% CI, 1.011, 7.628)).

Respondents who said that electricity, LPG, and biogas were more expensive were 3.27 times more likely to utilize traditional cooking energy sources as compared with those who said wood, charcoal, dung, and leaves were less expensive (AOR = 3.27 (95% CI, 1.05, 10.115)).

Households in Debre Markos town were paying about birr 190, interquartile range (IQR) (120, 255), for their monthly household cooking energy utilization. From this, respondents' median monthly budget for traditional cooking energy sources from their monthly income was birr 180, IQR (110, 250). Proportionally, 20.33% of their monthly income was budgeted for all cooking energy sources with a median of 20.33%, IQR (11.67%, 37.5%).

**When asked why many people of this town were not using electricity, for example, for cooking purpose, the expert from EEPCO stated that*“…usually we are informing our customers about the right time to use electricity for cooking on the back side of our collection bills. But, I think they still need further information as how electricity is cheaper source of cooking than others.”*

**On the other hand, a 35-year-old woman replied,*“I have tried to cook with electricity many times. But, especially to bake Injera much power is required that makes it impractical on the day time.” *

**A 25-year-old woman when asked about this reported*“from the very beginning to use electricity for cooking purpose needs a special electric meter which is better than what we have currently.” *

Households whose monthly income was birr 1001 and below were 5.14 times more likely to utilize traditional cooking energy sources than those whose monthly income was above birr 1001 (AOR = 5.14 (95% CI, 1.129, 23.415)) (Table 4).

## 4. Discussion

In this study, traditional cooking energy utilization was 95% which is almost the same as the 95.8% of Ethiopian households' traditional cooking energy utilization [10]. On the other hand, traditional cooking energy utilization of the study area (95.3%) was found to be lower as compared with the national figure in 2012 (99%), where 77% was from firewood, 13% from animal dung, and 9% from crop residues [3, 9]. This can be attributable to the fact that participants of the present study were only from urban dwellers.

Ethiopian energy consumption based on the energy policy of Ethiopia as of May 2010 was predominantly based on traditional energy source (utilization of 94%) which is almost similar to the prevalence of this study finding [11]. This similarity could be due to cultural and policy similarities of the country at large.

Participants' knowledge about the harmful effects of smoke from traditional biomass fuels in this study was found to be much lower as compared with a study conducted in Costa Rica (81%). However, the respondents' knowledge about effects of smoke from cooking whether it causes cough or not according to this study (83.5%) was generally better than Costa Rica's counterparts (65%) [12]. This slight mismatch could be explained by the awareness differences between the two study areas.

Literature indicated that education of the respondents could play a pivotal role in the choice of energy source for cooking [12]. However, despite the fact that the majority (72.8%) of the study participants were educated, they used traditional cooking energy sources. This finding is consistent with findings in studies conducted in southwestern Nigeria [13]. However, the present study showed that significant proportion of respondents (85.8%) relied on firewood as their primary cooking energy source against study results in Nigeria, where the respondents' primary cooking energy source was charcoal [13]. This difference in choice between Ethiopians and Nigerians could be a function of geographical variation in the level of urbanization, living standard, and climate and socioeconomic and cultural factors [13]. In addition to this, participants in the reference study being from rural and urban dwellers could be one reason for the observed discrepancy.

In this study, income was found to have statistically significant association with traditional cooking energy utilization, which is relatively the same as the study conducted in the United Kingdom concerning the spending pattern of the urban poor on cooking energy [6].

Knowledge of respondents had strong positive effects on traditional cooking energy utilization; about 42% of the respondents reported that firewood smoke affects health. This finding is much lower as compared to the result of studies conducted in southern Philippines [14]. But, almost a similar finding to this study was reported by a study conducted in Catembe, Mozambique, that compared respondents' knowledge about the effects of smoke from charcoal and firewood on the environment (54% and 53%), respectively [15]. This similarity may be partly explained by the fact that people became aware of the existence of global climate change and its consequences. About 355 (84.2%) of the respondents of this study had separate private kitchens which appeared to be higher than findings from other studies conducted in Ethiopia such as Jimma, where only 27.5% of respondents had separate private kitchens [16]. This mismatch could be due to study period differences and study subject differences in the two researches.

On the other hand, availability of cooking energy sources showed statistically significant association with traditional cooking energy utilization as it had no significant association in other previous studies [12, 16]. However, this finding is contrary to the fact that wood becomes less available through time which leads to climate change, soil erosion, and severe social implications [12].

The degree of expensiveness (expense perception) showed statistically significant association with traditional cooking energy utilization. This finding was in agreement with a study done in Canada [16]. This might be explained by the fact that lower income people are retailers such that they rely on easily accessible traditional biomass fuels despite their consequences.

The pretext participants, for not utilizing modern cooking energy sources other than their limited understanding, were perceiving the cost of modern cooking energy sources as being more expensive than the traditional ones. But, despite the fact that the monthly expenses for electricity consumption reported by the study participants included cost of lighting, it was still less in amount as compared with the monthly expense paid by respondents who paid for firewood consumption. This is because of the fact that 98 more respondents reported that they were paying more than birr 100 for firewood consumption as compared with nothing more than birr 100 for electricity. In addition to this, only 18 and 4 of the respondents reported that they were utilizing LPG and biogas with a monthly cost of birr 10 to birr 100 and birr 15 to birr 80, respectively, as compared with 52 more respondents who reported that they were paying more than birr 100 for their monthly expense of charcoal consumption. This finding clearly showed that most study participants were blindly reserving themselves from not utilizing modern fuels for cooking. The quantitative findings of this study were supported by qualitative findings which increase the strength of the study findings. Being of a cross-sectional study design, it may not clearly indicate causal effect relationship due temporal relationship.

In conclusion, traditional cooking energy utilization among households of Debre Markos inhabitants was found to be high. The major driving forces for this high utilization were wrong perception about cost of cooking energy sources, insufficient knowledge about the consequences of biomass utilization, limited household income, and wrong perception about the of availability of cooking energy sources. The implications of traditional cooking energy utilization on the community of Debre Markos town were poor health, ecological imbalance, and cost ineffectiveness. Therefore, understanding the utilization of traditional cooking energy sources and its correlates among households of Debre Markos town is the fundamental element of interventions for improving the health of the community, maintaining ecological balance, and minimizing cost of living.

Based on the findings of this study, the following recommendations were forwarded:

- in order to get our country, Ethiopia, developed clean cooking fuels, more efficient cooking technologies should be promoted and scaled up by the government as alternatives to traditional biomass fuels,
- government and nongovernment organizations should raise the awareness of the community about the health, cost, and environmental benefits of modern fuels,
- the community should be encouraged to use energy mix for making energy transition,
- more research is needed to measure the health, economical, and climatic impacts of energy interventions in regions where there is high dependence on traditional cooking energy sources like Debre Markos town.

## Figures and Tables

**Table 1:** Sociodemographic characteristics of study participants among households of Debre Markos town, northwest Ethiopia, May 2013.

Variable	Category	Number	Percent
Age	18–24	167	39.5
	25+	256	60.5
	Total	**413**	**100**

Religion	Orthodox	413	97.6
	Muslim	8	1.9
	Protestant	2	0.5
	Total	**423**	**100**

Marital status	Never married	53	12.5
	Married	273	64.5
	Divorced	66	15.6
	Widowed	31	7.3
	Total	**423**	**100**

Occupation	Government employee	105	24.8
	Merchant	96	22.7
	Self-employed	61	14.4
	Farmer	25	5.9
	Housewife	115	27.2
	Student	21	5
	Total	**423**	**100**

Education	Unable to read and write	115	27.2
	Read and write only	75	17.7
	Primary education	42	9.9
	Secondary education	108	25.5
	Technical and vocational	21	5
	Diploma and above	62	14.7
	Total	**423**	**100**

Family size	2–4	233	55.1
	4 and above	190	44.9
	Total	**423**	**100**

Household income	Up to birr 1001	257	60.8
	Birr 1001 and above	166	39.2
	Total	**423**	**100**

**Table 2:** Monthly budgets for each fuel type utilized among households of Debre Markos town, northwest Ethiopia, 2013.

Utilized fuel type	Monthly budget category	Number/percent
Wood	Birr 8–100	318 (75%)
	Birr 101–500	105 (25%)

Charcoal	Birr 5–75	233 (55%)
	Birr 76–300	290 (69%)

Animal dung	Birr 5–79	388 (92%)
	Birr 80–120	25 (6%)

LPG	Birr 10–50	17 (4%)
	Birr 51–100	12 (3%)

Biogas	Birr 0	419 (99%)
	Birr 15–80	4 (1%)

Electricity	10–75	29 (7%)
	76–100	7 (2%)

**Table 3:** Knowledge about the effects of traditional cooking energy utilization among households in Debre Markos town, northwest Ethiopia, May 2013.

Variable	Category	Number	Percent
Affects one's health	Knowledgeable	178	42
	Less knowledgeable	245	58

Affects the environment	Knowledgeable	207	49
	Less knowledgeable	216	51

Affects one's economy	Knowledgeable	313	74
	Less knowledgeable	110	26

**Table 4:** Correlates of traditional cooking energy utilization and each explanatory variable, Debre Markos, northwest Ethiopia, May 2013.

Traditional cooking energy utilization						
Explanatory variable	Category	Yes (%)	No (%)	COR (95% CI)	AOR (95% CI)	*P* value
Income	>1001	149 (35)	2 (1)	1.00	1.00	
	≤1001	254 (60)	18 (4)	5.28 (1.208, 23.072)	5.14 (1.129, 23.415)	0.024
Knowledge of effects on health	Yes	176 (41)	2 (0.5)	1.00	1.00	
	No	227 (54)	18 (4.5)	6.97 (1.598, 30.473)	7.56 (1635, 34.926)	0.01
Knowledge of effects on the environment	Yes	192 (45)	15 (4)	1.00	1.00	
	No	213 (50)	3 (1)	6.23 (1.797, 21.581)	6.68 (1.80, 24.385)	0.004
Expense perception	Electricity, biogas, LPG	204 (48)	15 (4)	2.93 (1.044, 8.204)	3.27 (1.057, 10.115)	0.04
	Wood, animal dung, leaves, charcoal	199 (47)	5 (1)	1.00	1.00	
Easily available cooking energy source	Electricity, biogas, LPG	100 (24)	10 (2)	1.00	1.00	
	Wood, animal dung, leaves, charcoal	303 (72)	10 (2)	3.03 (1.266, 7.491)	2.763 (1.011, 7.628)	0.04
